# TRIM56: a promising prognostic immune biomarker for glioma revealed by pan-cancer and single-cell analysis

**DOI:** 10.3389/fimmu.2024.1327898

**Published:** 2024-01-29

**Authors:** Bingcheng Wang, Zhihai Wang, Yuchen Li, Zehan Shang, Zihao Liu, Hao Fan, Rucai Zhan, Tao Xin

**Affiliations:** ^1^ Department of Neurosurgery, Shandong Provincial Qianfoshan Hospital, Shandong University, Jinan, China; ^2^ Department of Neurosurgery, Shandong Provincial Hospital, Shandong University, Jinan, China; ^3^ Department of Neurosurgery, The First Affiliated Hospital of Shandong First Medical University and Shandong Provincial Qianfoshan Hospital, Shandong Medicine and Health Key Laboratory of Neurosurgery, Jinan, China; ^4^ Department of Neurosurgery, Jiangxi Provincial People’s Hospital Affiliated to Nanchang University, Nanchang, China; ^5^ Medical Science and Technology Innovation Center, Shandong First Medical University and Shandong Academy of Medical Sciences, Jinan, China

**Keywords:** glioma, prognosis, tumor microenvironment, immune response, immune checkpoint, biomarker

## Abstract

Tripartite-motif 56 (TRIM56) is a member of the TRIM family, and was shown to be an interferon-inducible E3 ubiquitin ligase that can be overexpressed upon stimulation with double-stranded DNA to regulate stimulator of interferon genes (STING) to produce type I interferon and thus mediate innate immune responses. Its role in tumors remains unclear. In this study, we investigated the relationship between the expression of the TRIM56 gene and its prognostic value in pan-cancer, identifying TRIM56 expression as an adverse prognostic factor in glioma patients. Therefore, glioma was selected as the primary focus of our investigation. We explored the differential expression of TRIM56 in various glioma subtypes and verified its role as an independent prognostic factor in gliomas. Our research revealed that TRIM56 is associated with malignant biological behaviors in gliomas, such as proliferation, migration, and invasion. Additionally, it can mediate M2 polarization of macrophages in gliomas. The results were validated *in vitro* and *in vivo*. Furthermore, we utilized single-cell analysis to investigate the impact of TRIM56 expression on cell communication between glioma cells and non-tumor cells. We constructed a multi-gene signature based on cell markers of tumor cells with high TRIM56 expression to enhance the prediction of cancer patient prognosis. In conclusion, our study demonstrates that TRIM56 serves as a reliable immune-related prognostic biomarker in glioma.

## 1 Introduction

Glioma constitutes the predominant fraction of malignant brain tumors that manifest within the central nervous system among adults (1). This condition is distinguished by its marked heterogeneity and swift clinical advancement, resulting in a generally unfavorable prognosis for glioma patients (2). Malignant diffuse gliomas are categorized into grades II to IV, with grade II and III gliomas commonly referred to as LGG, and grade IV gliomas commonly referred to as GBM, based on their histological characteristics (3). Surgical resection, radiotherapy, and chemotherapy represent the principal therapeutic modalities employed for the management of malignant gliomas (4).

Nevertheless, despite the aforementioned treatments, the median survival duration for individuals with glioma remains below 15 months, with a 5-year survival rate not surpassing 5% (5). Consequently, the exploration of novel treatment alternatives continues to be a crucial aspect of glioma research. Immunotherapy has consistently garnered significant attention in the realm of glioma treatment, with particular emphasis on targeting the immune microenvironment of glioma as a pivotal avenue (6).

The tumor microenvironment (TME) plays a crucial role in the pathogenesis and therapeutic management of glioma, harboring a substantial population of immune cells that engage in intricate interactions with tumor cells, thereby influencing tumor cell proliferation, invasion, and other related processes (7). Notably, the infiltration of specific immune cell subsets, particularly the immunosuppressive M2 macrophages, is widely regarded as a prognostic indicator of unfavorable outcomes in glioma cases (8).

Based on prior research findings, it has been observed that M2 macrophages, in contrast to M1 macrophages, possess immunosuppressive properties and are capable of releasing diverse cytokines and signaling molecules to facilitate glioma invasion and proliferation (9). The tumor gene landscape is an important determinant of changes in TME composition and function.

Studying the role of key immune genes in the interaction network between immune cells and tumor cells will enhance our comprehension of the potential molecular mechanisms of tumor immune microenvironment on tumor progression from low grade to high grade, invasion and proliferation, so as to find better immunotherapy programs (10).

Tripartite-motif (TRIM) 56 (TRIM56) is a member of the TRIM family, and most members of the TRIM family possess E3 ubiquitin ligase activity and mediate ubiquitination of their corresponding substrates (11). In a previous study, TRIM56 was shown to be an interferon-inducible E3 ubiquitin ligase that can be overexpressed upon stimulation with double-stranded DNA to regulate stimulator of interferon genes (STING) to produce type I interferon and thus mediate innate immune responses (12, 13). Type I interferon has been shown to be an anti-tumor cytokine, which can inhibit tumor growth directly by acting on tumor cells or indirectly by acting on immune cells (14). As a factor that promotes interferon production, we hypothesized that TRIM56 plays an important role in tumor immunity (15). For example, in multiple myeloma, TRIM56 can inhibit cell proliferation and mediate apoptosis (16). TRIM56 can also inhibit the malignant development of hepatocellular carcinoma by targeting RBM24 and inactivating Wnt signaling (17). However, the role of TRIM56 in tumors is still unclear, and its expression and functional changes in gliomas are rarely reported.

In our study, we will use bioinformatics analysis combined with basic experiments based on TCGA, CGGA and GEO databases to explore the relationship between the expression level of TRIM56 and pan-cancer prognosis, and to confirm the expression pattern, potential function and diagnostic value of TRIM56 in glioma.

## 2 Materials and methods

### 2.1 Single-cell RNA sequencing analysis

The single cell dataset GSE182109 was downloaded from the GEO database, which contained a total of 2 LGG patients and 16 GBM patients (18). Low-quality single cells with less than 500 expressed genes or more than 20% mitochondrial transcripts or more than 50% ribosomal transcripts were removed. We identified potential single-cell doublets using the R package “DoubletFinder” with an expected doublets rate of 7.5%. After removing low quality and double cells, single cells were normalized and clustered using the R package “Seurat” and batch corrected using “Harmony”. Single-cell gene expression counts were normalized by library size and log2 transformation. We applied principal component analysis using the top 2000 most variable genes in the dataset to reduce the dimensionality of the data. The calculated principal components were batch corrected for differences between patients using “Harmony”. Cluster specific marker genes were identified using the function of “FindMarkers” of the R package “Seurat”, and genes specifically expressed in each cluster were detected to determine the cell type.

### 2.2 RNA sequencing and tissue microarray data collection

A total of 155,776 RNA-Seq gene expression profiles and clinical data of tumor and normal tissue samples were obtained from the UCSC Xena database, including information of tumor samples from TCGA database and information of normal samples from TCGA and GTEx databases. In addition, mRNA expression and clinical data of 325 glioma patient samples were obtained from the mRNAseq_325 dataset of the CGGA database. The molecular classification information of glioma was derived from cBioPortal, mainly including clinical information such as IDH and 1p/9q classification of TCGA samples (19, 20). Tissue microarray N109Ct01 was obtained from Sinochem Guanghua (Xi ‘an) Intelligent Biotechnology Co., LTD., which included 109 cases of glioma and their corresponding paracancerous brain tissues.

### 2.3 Immune characteristics analysis

Single sample gene set enrichment analysis (ssGSEA) in the “gsva” R package was used to determine the immune cell infiltration score using the marker gene set of immune cells. We used the R package “MCPcounter” and the R package “ESTIMATE” to quantify the levels of immune cell and stromal cell infiltration. The infiltration levels of 22 immune cells were calculated by the “CIBERSORT” algorithm.

### 2.4 Functional enrichment analysis

The differentially expressed genes between the two groups were obtained using the R package DESeq2 and the Wilcoxon rank-sum test to identify potential molecular alterations between the TRIM56 high and low expression groups. The screening criteria for differentially expressed genes (DEGs) were limited to genes with an absolute log value of fold change greater than 2 and an adjusted p-value less than 0.05. The differentially expressed genes were analyzed by Gene ontology (GO) and Kyoto Encyclopedia of Genes and Genomes (KEGG) using R package ‘cluster profile’ to explore their enriched biological functions and pathways. At the same time, gene set enrichment analysis (GSEA) was performed using the gene sets downloaded from the MSigDB website to explore the differences between high and low expression groups.

### 2.5 Cell culture and transfection

Glioma cell lines LN229, A172, U251, GL261 and macrophage cell lines RAW264.7 were obtained from Shanghai Institutes for Biological Sciences Cell Resource Center (Shanghai, China), and we obtained normal human astrocytes (NHA) from Sciencell Research Laboratories (Carlsbad, CA, USA). All the cell lines were cultured with DMEM medium mixed with10% fetal bovine serum at 37° in 5% CO2 cell incubator. Cells were cultured in six-well plates (NEST Biotechnology Co., Ltd., Wuxi, China) and TRIM56 was transfected into the glioma cells with Lipofectamine 2000.

The TRIM56 overexpression plasmid sequence was used as follows:

Human TRIM56 overexpression:5′-GCAGCAGAATAGTGTGGTAAT-3′;

Mouse TRIM56 overexpression: 5′-CGCCTTTAAGACCAACTTCTT-3′.

### 2.6 Western blot

The cells were lysed with a mixture of protease inhibitors, phosphatase inhibitors, and RIPA lysis buffer, an analysis of the protein concentration was performed using the BCA protein quantification kit. β-actin acted as loading controls for cytoplasmic and nuclear protein extracts, and the antibodies in western blot as follows: Anti-β-actin (1:50,000 dilution, 66009-1-Ig; Proteintech, Wuhan, China); Anti-TRIM56 (1:10,000 dilution, ab154862; abcam, Shanghai, China); Horseradish peroxidase (HRP)-conjugated secondary antibodies (1:3,000 dilution, M21003S; Abmart, Shanghai, China).

### 2.7 Cell scratch assay

LN229 and A172 cells transfected with TRIM56 overexpression plasmid were placed in a 6-well plate at the density of 1 × 10^6^ cells per well. 24 hours after transfection, the cells were scratched with a pipette, and the cells were observed under microscope after PBS washing and repeated observation after 12 hours of culture.

### 2.8 Ethynyldeoxyuridine assay

EdU detection kit (RiboBio, Guangzhou, China) was used to detect the DNA replication which represents proliferation activity of the cells. The ratio of the number of cells stained with EdU to the number of cells stained with Hoechst-33342 was used to represent EdU incorporation rate.

### 2.9 Cell cycle analysis

LN229 and A172 cells were collected 48 hours after transfecting TRIM56 overexpression plasmid, then the cells were washed twice with phosphate-buffered saline (PBS), and fixed with 70% precooled ethanol at 4° overnight. The cells were stained with a cell cycle kit (Beyotime, Shanghai, China) and performed by flow cytometry for cell cycle distribution, then analyzed by Modfit 5.0 software.

### 2.10 Immunohistochemistry

Immunohistochemical analysis of paraffin-embedded glioma tissue sections was performed. The staining intensity was divided into 4 grades, with 0 representing negative, 1 representing weak, 2 representing medium and 3 representing strong. We used H-score method to obtain TRIM56 protein expression for semi-quantitative score. H-score = (percentage of cells stained with weak intensity ×1) +(percentage of cells stained with medium intensity ×2) +(percentage of cells stained with strong intensity ×3). The H-score, measured by the software Aipathwell, ranges from 0 to 300, and the scores of repeated samples were averaged. IHC: TRIM56 (1:1000) abcam, Shanghai, China.

### 2.11 Quantitative real-time PCR

RNA was extracted from cells by Trizol reagent. qRT-PCR was implemented on a Real-Time PCR system using the SYBR. GAPDH was used as endogenous control, and 2-ΔCt method was used for comparative quantitative analysis. The sequence of primers was as follows:

TRIM56, 5’-TTCTTCGTCAATGGGCTGCT-3’ (forward) and 5’-AAGTCATCGGCACAGTCCAG -3’ (reverse).

GAPDH, 5’-GAGAAGTATGACAACAGCCTCAA-3’ (forward) and 5’-GCCATCACGCCACAGTTT-3’ (reverse).

### 2.12 Flow cytometry

Firstly, TRIM56 overexpressed glioma cell lines were co-cultured with macrophage cell lines using Transwell (Corning, USA). Subsequently, macrophages were individually stained with PE-conjugated CD86 antibody (eBioscience, USA) and APC-conjugated CD163 antibody (eBioscience, USA). To eliminate interference from intrinsic cellular fluorescence, gating was applied to unstained cells during data analysis (control M1 and control M2). The experiments were conducted using the CytoFlex3 instrument (Beckman Coulter), and the results were analyzed using CytExpert software.

### 2.13 Tumor xenograft model

In the orthotopic xenografts, transplant 5 × 10^5^ overexpressing TRIM56 LN229 cells expressing firefly luciferase into the frontal lobe of 4-week-old male BALB/c nude mice. BALB/c nude mice (males) were purchased from Beijing Vital River Laboratory Animal Technology Co., Ltd. (Beijing). Bioluminescence imaging (IVIS Spectrum *in vivo* imaging system, PerkinElmer, USA) was used to monitor tumor growth at 7and 14 days.

### 2.14 Immunofluorescence staining

The paraffin sections were meticulously prepared by Servicebio (Wuhan, China), in accordance with prescribed procedures for antigen retrieval. Following a 30-minute blockade with 3% BSA, the primary antibody (Anti-CD163, 1:500, GB113751, Servicebio) was applied and allowed to incubate overnight at 4°C within a controlled humidified environment. Subsequently, the corresponding secondary antibody [Goat Anti-Mouse IgG (Alexa Fluor 594), 1:400, Jackson, 115-585-003] was administered, and cell nuclei were counterstained with DAPI (Thermo Fisher, D1306, USA). Fluorescence signals were then meticulously detected utilizing laser confocal microscopy (ZEISS LSM 800).

### 2.15 Statistical analysis

Gene expression data were standardized to improve the accuracy of the study. Statistical differences were assessed with the use of t-tests or Wilcoxon rank-sum tests, if deemed appropriate. Results are presented as the mean ± standard deviation (SD) for each group. The “glmnet” R package was used for variable selection and shrinkage using the LASSO algorithm. Regression was performed with the normalized expression matrix of the putative cell marker gene set as the independent variables, and the response variables were overall survival and patient status in the TCGA cohort. The penalty parameter λ for the signature was calculated by ten-fold cross validation of the minimum criterion. Wilcoxon rank sum test was used for pairwise comparison between groups. Spearman test was used to calculate the correlation between different variables, and the R package “circlize” and “heatmap” were used for visualization. Univariate Cox regression analysis was used for survival analysis to obtain the corresponding hazard ratios and p values. Kaplan-Meier curve was used to analyze the survival related information of high-risk group and low-risk group. The R package “maftools” was used to visualize the mutation of genes in the high-risk group and the low-risk group, and to describe the tumor mutation burden (TMB) between different groups. Other R packages such as “ggplot2” were also used for visualization of the analysis results. A p-value of less than 0.05 was considered to indicate a significant difference.

### 2.16 Drug sensitivity

The “proprophetic “R software package was used to calculate the half maximal inhibitory concentration (IC50) value of the two groups of glioma patients after multi-drug treatment to explore the drug sensitivity of the two groups of patients.

## 3 Results

### 3.1 Expression and prognosis of TRIM56 in pan-cancer

**Figure 1:**
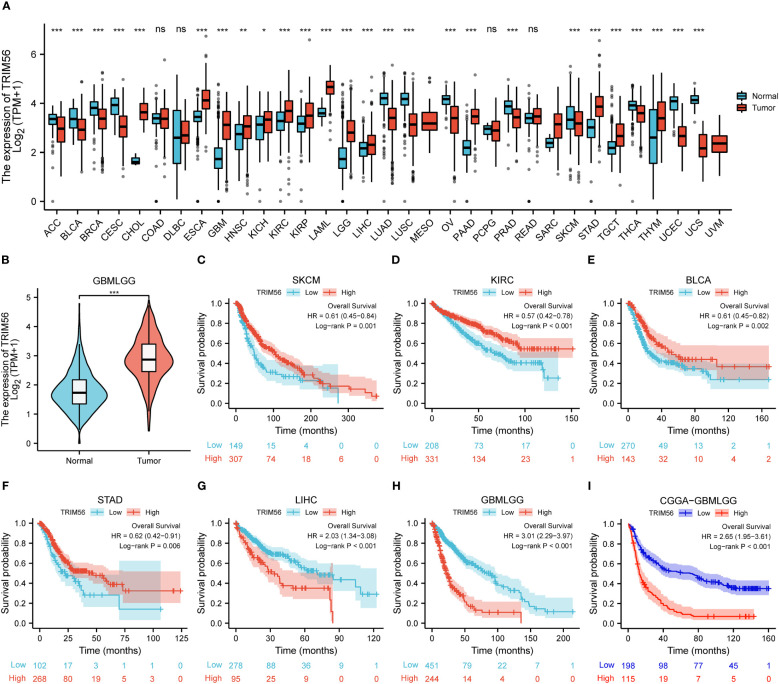
Expression and prognosis ofTRIM56 in pan-cancer. **(A, B)** The mRNA expression of TRIM56 in pan-cancer. The effect of TRIM56 expression on overall survival (OS) of SKCM **(C)**, KIRC **(D)**, BCLA **(E)**, STAD **(F)**, LIHC **(G)**, glioma in TCGA dataset **(H)** and glioma in CGGA dataset **(I)** was analyzed by Kaplan-Meier method. p < 0.05, * p < 0.05, **p < 0.01, ***p < 0.001. ns, not statistically significant.

In order to explore the expression of TRIM56 on a pan-cancer scale, we used TCGA database and GTEx database to analyze the expression level of TRIM56 in tumor tissues and normal tissues. In detail, TRIM56 expression was significantly lower in 12 cancer types and significantly higher in 13 cancer types than in normal tissues (**Figures 1A, B**). Next, we investigated the prognostic value of TRIM56 expression in the aforementioned 25 cancers. Kaplan-Meier method was used to analyze Overall Survival (OS) of patients from the TCGA database. Notably, TRIM56 expression was significantly correlated with OS in patients with a total of 6 cancer types, including KIRC, SKCM, STAD, BLCA, LIHC, GBMLGG (**Figures 1C–H**). Interestingly, TRIM56 played a protective role in four cancer types, including KIRC, SKCM, STAD, BLCA, however, it is a detrimental factor for two cancers, including GBMLGG and LIHC. We selected GBMLGG as a representative of unfavorable factors to conduct a detailed study. Then we selected the CGGA database for verification and obtained the same conclusion (**Figure 1I**).

### 3.2 Expression pattern and diagnostic value of TRIM56 in gliomas

**Figure 2:**
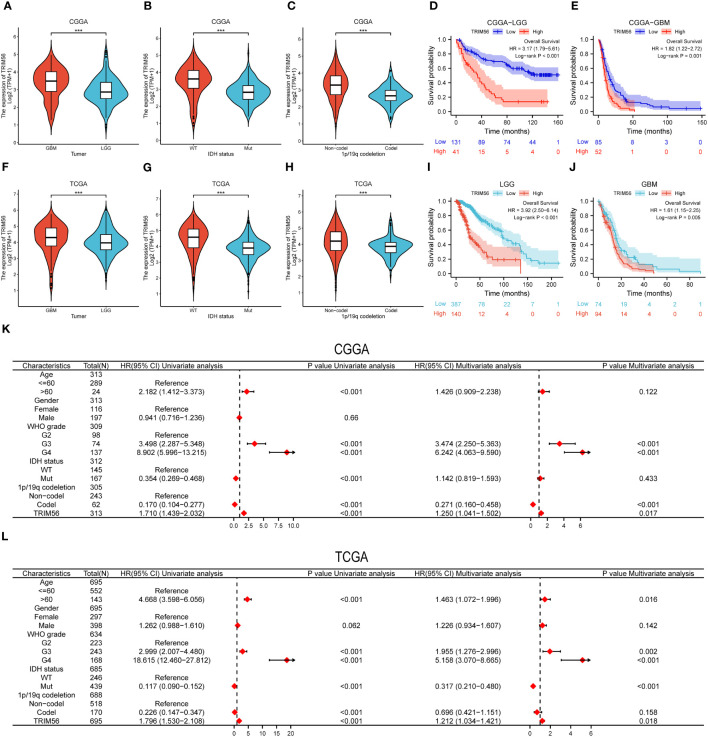
Expression pattern of TRIM56 in different molecular subtypes and its diagnostic value. **(A, F)** TRIM56 was significantly increased in GBM in TCGA and CGGA datasets. **(B, G)** TRIM56 was significantly increased in isocitrate dehydrogenase (IDH)-wildtype glioma in TCGA and CGGA datasets. **(C, H)** TRIM56 was significantly upregulated in the 1p/19q non-codel gliomas in TCGA and CGGA datasets. The effect of TRIM56 expression on OS of LGG **(D)**, GBM **(E)** in CGGA dataset and LGG **(I)**, GBM **(J)** in CGGA dataset was analyzed by Kaplan-Meier method. **(K, L)** Univariate and multivariate Cox analysis of clinic-pathologic characteristics in glioma based on TCGA and CGGA datasets. ***p < 0.001.

**Figure 3:**
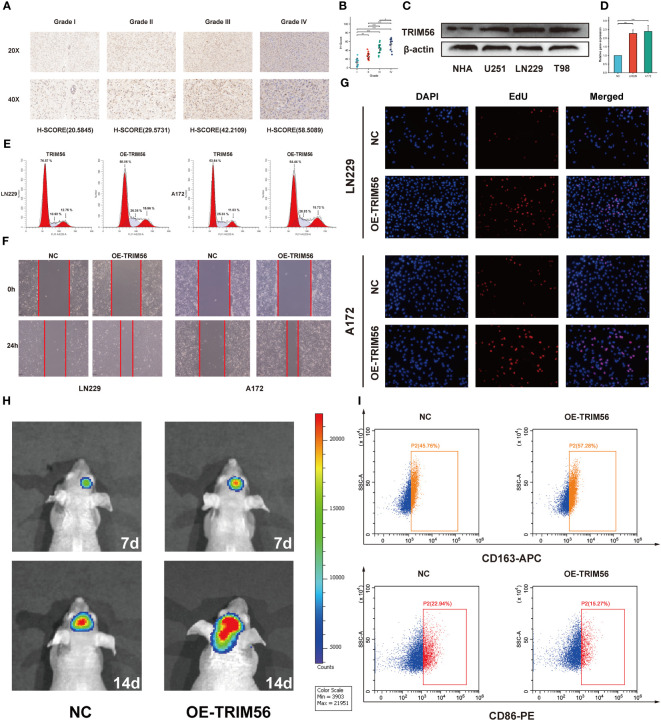
Validation of TRIM56 expression in glioma and the effect of TRIM56 overexpression in glioma cells on tumor proliferation, invasion, cell cycle and macrophage polarization. **(A)** Immunohistochemical analysis of TRIM56 in glioma. **(B)** The staining of TRIM56 was scored based on the H-score system. **(C)** Expression levels of TRIM56 protein in normal human astrocytes and glioma cell lines. **(D)** Real time PCR detected the efficiency of TRIM56 overexpression in LN229 and A172 cells. **(E)** Flow cytometry was used to detect the cell cycle. **(F)** Scratch test was used to determine the cell migration. **(G)** Ethynyldeoxyuridine (EdU) Assay analysis was preformed to determine the cell proliferation. **(H)** Situ tumorigenesis assay was used to determine the cell proliferation. **(I)** The effect of TRIM56 overexpression in glioma on macrophage polarization was detected by flow cytometry. * p < 0.05, **p< 0.01, ***p < 0.001.

To confirm the expression pattern of TRIM56 in gliomas, RNA-seq data of LGG and GBM from TCGA database were analyzed, and data from CGGA database were used for validation. GBM showed higher TRIM56 expression levels compared to LGG in TCGA database, and consistent results were obtained after validation using CGGA database (**Figures 2A, F**). IDH1 and IDH2 gene mutations and co-deletion of chromosome 1p and 19q are two important diagnostic factors for the prognosis of glioma patients (21, 22). In order to investigate the molecular expression pattern of TRIM56, we evaluated the expression levels of TRIM56 in IDH wild-type (WT) and IDH mutant (Mut) gliomas and in 1p/19q co-deletion and without 1p/19q co-deletion. Utilizing RNA-seq data from CGGA and TCGA, TRIM56 expression was significantly increased in IDH wild-type gliomas (**Figures 2B, G**) and gliomas without 1p/19q co-deletion (**Figures 2C, H**). We then used the KM method to predict whether TRIM56 was significantly associated with overall survival (OS)in LGG and GBM, and we found that high expression of TRIM56 in both GBM (**Figures 2E, J**) and LGG (**Figures 2D, I**) predicted significantly shorter OS in both CGGA and TCGA databases. We also used univariate and multivariate Cox regression to determine whether TRIM56 expression could serve as an independent prognostic factor in gliomas. In addition to TRIM56, we included common clinical glioma diagnostic factors such as age, gender, grade, IDH mutation status, and 1p/19q co-deletion (**Figures 2K, L**). Interestingly, in multivariate regression, after adjusting for clinical factors that had a significant effect on prognosis in univariate regression, TRIM56 expression was still an independent predictor in both databases. In addition, we verified the expression level of TRIM56 in different grades of gliomas by immunohistochemistry (IHC) of human glioma tissues, and the results showed that TRIM56 protein expression was higher in high-grade gliomas (**Figures 3A, B**). Furthermore, the detection of TRIM56 expression in various glioma cell lines was conducted via Western blot analysis, revealing that the expression levels of TRIM56 were significantly elevated in glioma cell lines compared to normal human astrocytes (**Figure 3C**).

### 3.3 TRIM56 immune infiltrating correlation in glioma

**Figure 4:**
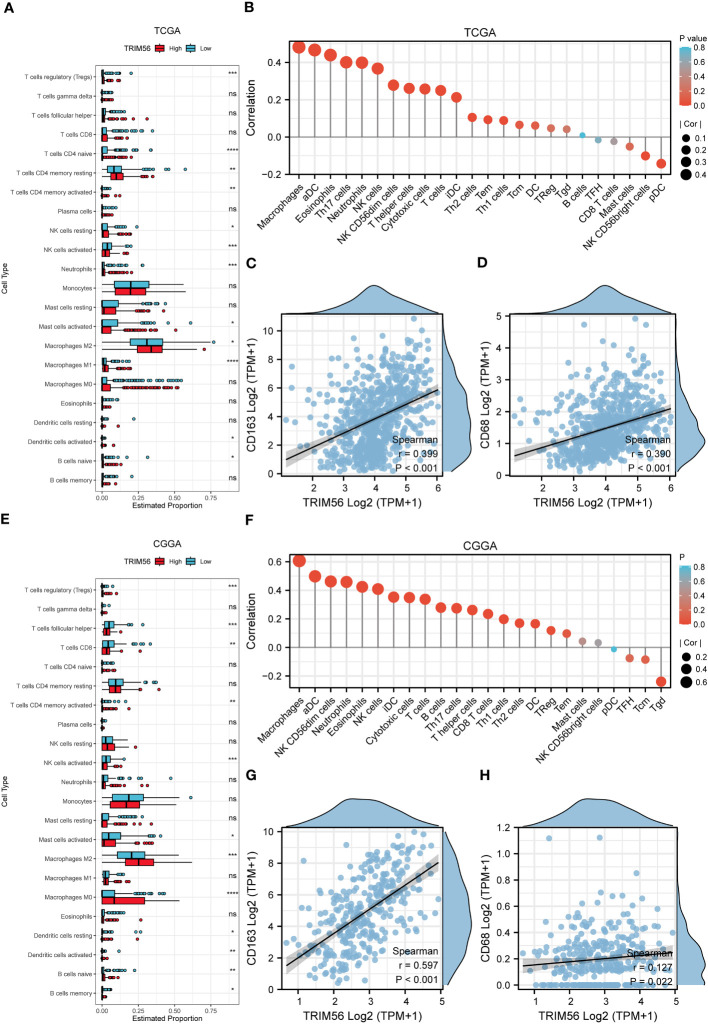
The landscape of immune infiltration in glioma with different expressions of TRIM56. **(A, E)** Box plots showed differential immune cell infiltration status with different expressions of TRIM56. The statistical differences of two groups were compared by CIBERSORT through TCGA and CGGA datasets. **(B, F)** The ssGSEA analysis was used to calculate the correlation between immune cells and TRIM56 expression levels in glioma. Correlation of TRIM56 expression with M2 macrophages **(C, D, G, H)** biomarkers in glioma. * p < 0.05, **p< 0.01, ***p < 0.001, ****p < 0.0001. ns = not statistically significant.

Tumor immunity is mainly mediated by immune cells in the tumor immune microenvironment (23, 24). Therefore, we explored the potential association between TRIM56 and glioma immune infiltration. We determined whether TRIM56 expression was associated with immune cell infiltration in gliomas by calculating the relationship between TRIM56 expression level and the marker genes of each immune-infiltrating cell using the ssGSEA algorithm (**Figures 4A, E**). TRIM56 expression had the highest correlation with the level of immune infiltration of macrophages in glioma, and we obtained the same results in TCGA and CGGA databases. We then used the CIBERSORT method to validate and calculate each cell subtype (**Figures 4B, F**). The results showed that TRIM56 expression level was positively correlated with M2 macrophage infiltration level in glioma. It is well known that M2 macrophages play a crucial role in glioma immunosuppression, tumor progression and metastasis, and have a significant adverse effect on the prognosis of glioma patients. Combining the above two analyses, we observed a significant difference in the expression of M2 macrophages between TRIM56 high and low expression groups in gliomas. We further calculated the correlation between M2 macrophage biomarkers CD163 (**Figures 4C, G**) and CD68 (**Figures 4D, H**) and TRIM56, and the results further confirmed the above results. TRIM56 expression was most closely related to M2 macrophages in glioma. By co-culturing M0 macrophages with glioma cells and TRIM56 overexpressing glioma cells, and verified by flow cytometry, we found that macrophages in TRIM56 overexpression group were significantly polarized to M2 macrophages compared with the control group (**Figure 3I**).

### 3.4 Enrichment analysis and the effects of TRIM56 on proliferation, migration and invasion of glioma

**Figure 5:**
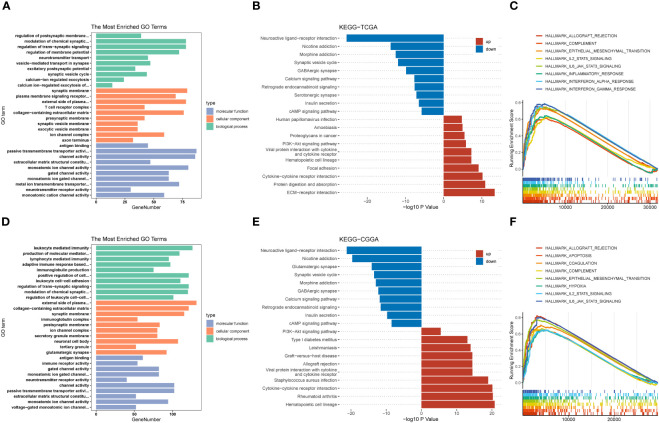
Potential functions of TRIM56. **(A, D)** Histogram of top 10 enriched GO terms of molecular function, cellular component and biological process. **(B, E)** Histogram of top 20 enriched KEGG pathways. **(C, F)** Gene set enrichment analysis (GSEA) of the top 8 enriched pathways in TRIM56-high expression phenotype from TCGA and CGGA datasets.

Next, we grouped the median expression of TRIM56 and performed differential analysis using R package DEseq2 to obtain differentially expressed genes (DEGs) in TCGA and CGGA databases. GO and KEGG analyses were used to explore the biological processes and pathways potentially associated with TRIM56 expression. The results of GO analysis showed that DEGs were significantly enriched in immune-related biological processes (**Figures 5A, D**). The PI3K−Akt signaling pathways were shown to be enriched in DEGs from both the TCGA and CGGA cohorts, according to KEGG pathway analyse (**Figures 5B, E**). Gene set enrichment analysis (GSEA) was performed for the two groups of differentially expressed genes using hallmark gene set of MSigDB (**Figures 5C, F**). The top eight highly expressed pathways were shown as follows. The “HALLMARK_IL6_JAK_STAT3_SIGNALING” gene set was significantly enriched in both TCGA and CGGA cohorts. Interestingly, we found that the “EPITHELIAL_MESENCHYMAL_TRANSITION” gene set, which is closely related to tumor invasion and proliferation (25), was more enriched in GBM than LGG, and TRIM56 was more strongly associated with tumor proliferation and invasion-related pathways in GBM (**Supplementary Figures 1A, B**). Clearly, compared with LGG, TRIM56 has a stronger association with tumor proliferation and invasion related pathways in GBM. In conclusion, TRIM56 is associated with glioma proliferation and invasion in addition to immunity. We constructed a TRIM56-overexpressing cell line to more specifically validate the enrichment analysis results, and verified the gene overexpression effect by qRT-PCR (**Figure 3D**). EdU assay and scratch assay were used to detect the effects of TRIM56 overexpression on the proliferation, migration and invasion of glioma cells. Overexpression of TRIM56 in LN229 and A172 cells significantly enhanced cell proliferation, invasion and migration (**Figures 3F, G**). TRIM56 regulation of cell cycle was detected by flow cytometry, and the results showed that overexpression of TRIM56 significantly increased the percentage of cells in S phase and G2/M phase, suggesting that overexpression of TRIM56 promotes the proliferation of glioma cells (**Figure 3E**). Additionally, we further demonstrated overexpression of TRIM56 promoted glioma progression under *in vivo* conditions by tumor xenograft model (**Figure 3H**). Additionally, immunofluorescence analysis was performed on the tumor xenograft model (**Supplementary Figure 2**). By comparing the changes of macrophage markers in glioma tissues, we further confirmed that overexpression of TRIM56 may affect the glioma immunosuppressive microenvironment by regulating macrophage polarization. Taken together, TRIM56 plays a key role in glioma proliferation, migration, invasion and cell cycle.

### 3.5 Immune functions and immune checkpoint analysis

**Figure 6:**
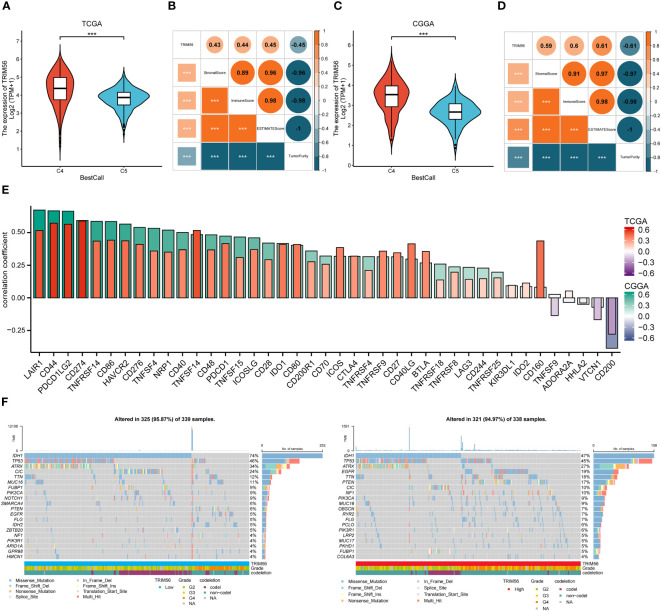
Correlation analysis between the expression of TRIM56 and immunophenotypes, immune checkpoint markers and tumor mutation burden (TMB). **(A, C)** TRIM56 expression was significantly overexpressed in the C4 subtype in TCGA and CGGA datasets. C4: the Immunologically Quiet subtype, C5: the Lymphocyte Depleted subtype. **(B, D)** Correlation of TRIM56 expression with immune infiltration level. **(E)** The correlation between TRIM56 and immune checkpoints in TCGA and CGGA datasets. **(F)** Tumor mutation landscape in TRIM56 high and low expression groups by oncoplots. ***p < 0.001.

In previous studies, tumors have been divided into six immune subtypes (C1 to C6), while gliomas mainly contain C4 (Immunologically Quiet) and C5 (Lymphocyte Depleted) subtypes, which represent different immune microenvironments (26). Simultaneity has different effects on the prognosis of patients, and C4 has a worse prognosis, we used the R package “ImmuneSubtypeClassifier” to calculate the expression level of TRIM56 in these two subtypes. The results showed that the expression level of TRIM56 in C4 was significantly higher than that in C5 in TCGA and CGGA databases (**Figures 6A, C**). Moreover, we used ESTIMATE methods were used to investigate the relationship between TRIM56 expression and the purity of tumor tissue, we could find that TRIM56 in glioma was significantly negatively correlated with tumor purity (**Figures 6B, D**). There was a significant positive correlation between TRIM56 and immune score, indicating that TRIM56 was mainly expressed in non-tumor cells, including stromal cells and immune cells. We also calculated the correlation between TRIM56 expression level and tumor purity in LGG and GBM, respectively. Clearly, the correlation between TRIM56 expression level and tumor purity in GBM was higher than that in LGG (**Supplementary Figures 3A, B**). In addition, the spearman method was used to calculate the correlation between TRIM56 expression level and immune checkpoint inclusion in gliomas. As shown in the figure, TRIM56 was positively correlated with most of the immune examination sites. Combined with TCGA and CGGA cohort, TRIM56 was most closely correlated with LAIR1, CD44, PDCD1LG2 and CD274 (**Figure 6E**), and oncoplots were used to display the tumor mutation gene microlandscape of TRIM56 high expression group and low expression group (**Figure 6F**). The top ten genes with the highest proportion of mutations in both sets are presented.

### 3.6 Single-cell analysis of TRIM56 in gliomas

**Figure 7:**
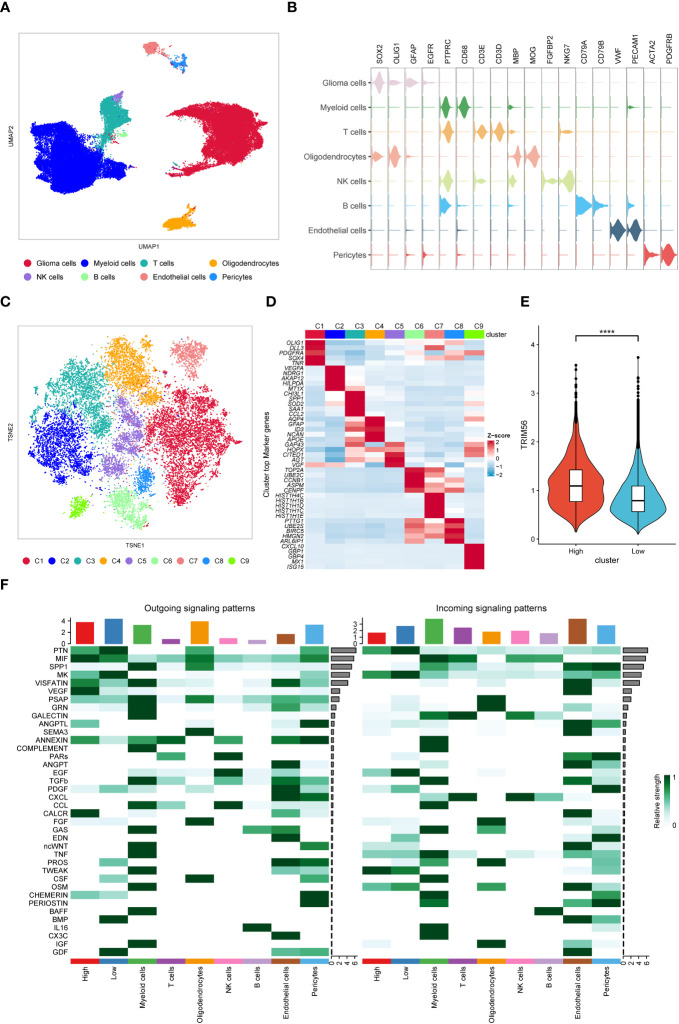
Single-cell analysis of TRIM56. **(A)** UMAP projections of single cells showing the composition of different cell types in human gliomas. **(B)** Violin plot showing marker gene expression for different cell types. **(C)** TSNE projections of single cells showing the composition of different tumor cell subsets. **(D)** Top 5 differentially expressed genes in clusters, ranked by FDR, are shown in the heatmap. **(E)** The expression level of TRIM56 in the high expression subgroup was significantly higher than that in the low expression subgroup. **(F)** Cell-to-cell communication was analyzed in TRIM56 high and low expression subsets with other cells. ****p < 0.0001.

In order to better study the effect of TRIM56 high expression glioma cells on the tumor microenvironment, we downloaded the single cell data of GES182109 for further study. After data cleaning and reduction and grouping, the TRIM56 positive cells were divided into glioma cells (expressing SOX2, OLIG1, GFAP, and EGFR), myeloid cells (mainly macrophages and microglia, expressing PTPRC and CD68), T cells (expressing PTPRC, CD3E, and CD3D), oligodendrocytes (expressing MBP and MOG), NK cells (expressing PTPRC, FGFBP2, and NKG7), B cells (expressing PTPRC, CD79A, and CD79B), endothelial cells (expressing VWF and PECAM1), and pericytes (expressing ACAT2 and PDGFRB) according to specific cell markers (**Figures 7A, B**). Then we extract the tumor cells again after group-dividing dimension reduction is divided into nine subgroup (C1 to C9) (**Figure 7C**). We found that the expression of TRIM56 in C2 subgroup was significantly higher than that in other subgroups (**Supplementary Figure 4**), which was defined as TRIM56 high expression tumor cell subgroup, and the remaining cell groups were defined as low expression tumor cell subgroup (**Figures 7D, E**). Then we used R package “Cellchat” to evaluate the cellular communication patterns of the two tumor subsets to other cells in the tumor microenvironment (27), and found that compared with TRIM56 low expression subset, TRIM56 high expression subset could act on macrophages/microglia and endothelial cells by overexpressing secreted proteins MIF and VEGF respectively (**Figure 7F**).

### 3.7 The construction of the TRIM56-related genes signature

**Figure 8:**
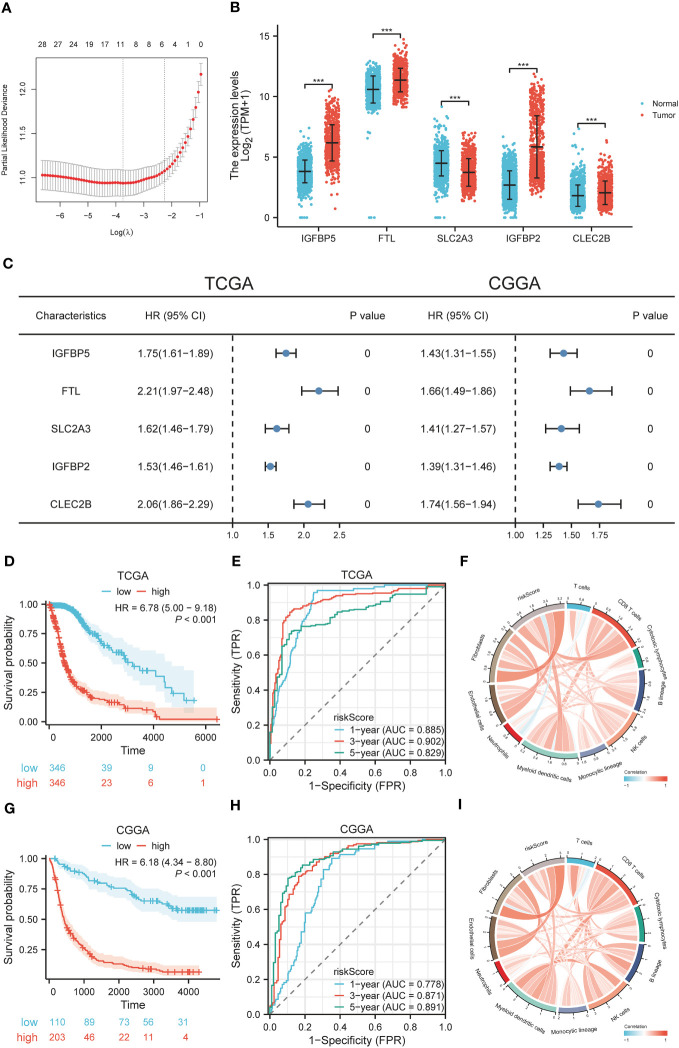
Construction and diagnostic value of gene signatures. **(A)** LASSO algorithm determines the genes and their coefficients for constructing the gene signature. **(B)** Expression levels of selected genes in tumor and normal tissues. **(C)** Univariate Cox analysis of selected genes in glioma based on TCGA and CGGA datasets. **(D, G)** The effect of risk scores on OS in TCGA and CGGA datasets. **(E, H)** The predictive value of risk scores in TCGA and CGGA datasets by ROC curve analysis. **(F, I)** Correlation between risk scores and cellular infiltration in the tumor microenvironment. ***p < 0.001.

To further evaluate the prognostic value of TRIM56 high expression tumor cells in glioma, we used the “Findmarkers” function in the R package “Seurat” to obtain marker gene sets of TRIM56 high expression subgroups with logFC greater than 1 and corrected p value less than 0.05 as screening criteria. Stepwise multivariate Cox regression analysis was included, and 5 genes were finally obtained for lasso cox regression, and a prognostic model was constructed based on the λ value of the least variable (**Figure 8A**). The expression levels of these five genes were significantly different between normal and tumor tissues (**Figure 8B**). The median risk score in the TCGA dataset was used as the grouping criterion for the CGGA dataset to validate the model. Combined with CCGA and TCGA databases, the high expression of these 5 genes had a significant adverse effect on the prognosis of patients (**Figure 8C**). The high-risk group was positively correlated with high tumor grade, IDH wild type, 1p/9q non-co-deletion, and age (**Supplementary Tables 5A, B**).

### 3.8 Diagnostic value and drug susceptibility analysis of genes signature

**Figure 9:**
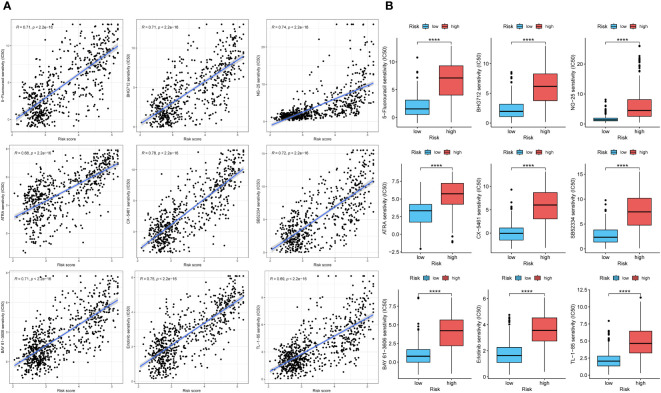
The analysis of curative effect of anti-tumor drugs. **(A, B)** The IC50 of indicated anti-tumor drugs in high and low risk score groups. ****p < 0.0001.

To explore the diagnostic value of the prognostic model, KM analysis showed that the high-risk score group had a poor prognosis (**Figures 8D, G**). A time-dependent ROC curve was used to evaluate the predictive performance of the risk score for OS (**Figures 8E, H**). The area under the curve (AUC) of 1, 3, and 5 years in TCGA training cohort was 0.885, 0.902, and 0.829, respectively, and the AUC of 1, 3, and 5 years in CGGA validation cohort was 0.778, 0.871, and 0.891, respectively. Using the R package “MCPcounter”, we found that the risk score was most correlated with fibroblasts in the tumor microenvironment (**Figures 8F, I**), and using the R package “proprophetic”, we identified nine antineoplastic agents that may benefit patients with a high-risk score (**Figures 9A, B**).

## 4 Discussion

Glioma is a severe malignant brain tumor that affects human health severely. LGG and GBM are components of glioma. Compared with LGG, GBM has a higher degree of malignancy, stronger invasion and higher fatality rate. The microscopic gene expression profiles of the two groups are different, and there are certain differences in the immune microenvironment of the two groups, which also lead to different roles of immune-related genes (28). This article has certain reference value for personalized treatment of glioma and the progress of glioma.

The role of TRIM56 has been elucidated in previous studies, but its role in tumors, although reported, is still unclear. Based on the clear correlation between TRIM56 and interferon production in previous studies, and the anti-tumor effect of interferon, we speculate that TRIM56 is a protective factor in most tumors. Pan-cancer analysis also confirmed our conjecture, but TRIM56 in glioma obtained a completely different result. OS analysis showed that high expression of TRIM56 in glioma indicated a significant poor prognosis of patients, which could not help but arouse our curiosity whether TRIM56 is a new immune marker in glioma. When applied to the TCGA and CGGA RNA-seq datasets, TRIM56 exhibited a significant association with glioma grades, IDH mutation status and codeletion of chromosomes 1p and 19q. The TRIM56 protein expression was also higher in high-grade gliomas, IDH wild-type gliomas and no 1p/19q codeletion gliomas. In addition, univariate and multivariate survival analyses also confirmed that TRIM56 was an independent prognostic factor.

Functional analyses such as GO and KEGG analyses were used to explain the underlying mechanisms of poor prognosis caused by TRIM56 overexpression. As analyzed by GO, TRIM56 expression was associated with the regulation of trans-synaptic signaling. In addition, many immune-related biological processes were enriched. The KEGG results suggested that TRIM56 played a role in the activation of PI3K-AKT pathway in glioma and “IL6-JAK-STAT3 signaling” gene set was enriched by GSEA analysis. Previous studies have shown that the above two pathways are involved in cell proliferation, metabolism, and immune regulation (29, 30), so we speculate that TRIM56 plays an important role in the proliferation of glioma. Then TRIM56 overexpression cell lines were constructed for EdU assay, wound healing assay, flow cytometry and tumor xenograft model *in vivo* to validate the enrichment analysis results more specifically.

In order to clarify the relationship between TRIM56 expression and immune infiltration-related cells, using CIBERSORT and ssGSEA algorithms, we can find that TRIM56 has a significant correlation with M2 macrophages in glioma, which is significantly correlated with poor prognosis of glioma. This partly explains the adverse effect of TRIM56 on the prognosis of patients. The above results were confirmed by co-culture of glioma cells and macrophages with flow cytometry.

In previous studies, gliomas were classified into six subtypes, C1 to C6. Glioma mainly contains C4 and C5 subtypes, the C4 subtype displays a more prominent macrophage signature, with low lymphocytic infiltrate and high M2 macrophage, which leads to an immunosuppressed TME and a poor outcome. C5 subtype fewer tumor-associated immune cells and better outcome. The results showed that gliomas consisted most of C4 (lymphocyte depleted) and C5 (immunologically quiet), C4 subtype had a worse prognosis than C5 subtype, and we observed that TRIM56 was significantly higher in C4 than C5 subtype. which revealed that TRIM56 possibly referred to a negative microenvironment. We further calculated the correlation between tumor purity and TRIM56 expression by estimate algorithm. We found that in gliomas, tumor purity was negatively correlated with TRIM56 content, but positively correlated with immunoscore. At the same time, our results also indicated that with the increase of tumor grade, the correlation between TRIM56 and tumor purity also increased, which meant that the expression of TRIM56 in glioma cells also increased. Therefore, we speculated that glioma cells with high expression of TRIM56 had a certain diagnostic value for the prognosis of glioma patients. Combining CGGA and TCGA databases, we found that TRIM56 was positively correlated with most of the immune checkpoints, and had the highest correlation with LAIR1, CD44, PDCD1LG2 and CD274. These studies demonstrated that TRIM56 in glioma is a potential predictive marker for ICH response.

Tumor mutation burden (TMB) is closely related to tumor immunotherapy (31). We further analyzed the relationship between TRIM56 and TMB. TMB was higher in TRIM56 high expression group, and the tumor mutation microlandscape of TRIM56 high expression group and low expression group showed that EGFR and PTEN mutation proportion in high expression group was significantly higher than that in low expression group. EGFR amplification and overexpression and PTEN mutation or loss are important factors leading to the progression of glioma (32, 33). Both EGFR and PTEN can act on the downstream PI3K−Akt pathway to promote glioma invasion and proliferation, which is consistent with previous results (34, 35).

To comprehensively assess the impact of TRIM56 on the glioma microenvironment, we utilized single-cell analysis. We identified a subgroup of glioma cells with elevated TRIM56 expression, showing increased secretion of VEGF and MIF proteins compared to other subgroups. VEGF influences tumor vascularization, fostering tumor growth and invasion by stimulating blood vessel proliferation (36, 37). Meanwhile, MIF affects macrophages/microglia, promoting an M2 immunosuppressive state, which contributes to a tumor immunosuppressive microenvironment and hinders glioma immunity (38, 39). In the previous study, it was observed that the expression of TRIM56 in tumor cells correlated positively with glioma grade. This finding suggests that high-grade gliomas tend to have a higher proportion of tumor cells with elevated TRIM56 expression, which contributes to the malignancy of the disease.

In order to better evaluate the effect of TRIM56 high expression subsets on the prognosis of glioma, we established gene signature for glioma using marker genes of TRIM56 high expression subsets. The prognostic signature included 5 genes (FDX1, LIAS, DLD, DLAT, PDHB, and MTF1). The expression level of genes integrated into signature in tumor tissues was significantly higher than that in normal tissues, and both had a negative impact on the prognosis of glioma patients. The corresponding risk scores were significantly correlated with a variety of clinical information of glioma, and time-dependent ROC curve was established to evaluate the predictive performance of risk scores. We found that the risk scores had high accuracy in predicting glioma survival. Using R package “MCPcounter”, the risk scores were most correlated with fibroblasts in the tumor microenvironment. These results suggest that TRIM56 overexpression in tumor subsets promotes the proliferation of tumor fibroblasts. We also used drug-susceptibility predictions to identify nine antineoplastic agents that were appropriate for the high-risk group.

In conclusion, this is the first study to investigate the gene expression, clinical features and biological functions of TRIM56 in gliomas by bioinformatics analysis techniques including bulk RNA-seq analysis and single-cell analysis. This study illustrates the molecular characteristics of TRIM56 in the progression of glioma to a certain extent, and reveals the expression of immune molecules and their corresponding functional changes during the progression of glioma. We constructed the gene signature of TRIM56 high expression subsets, which better predicted the prognosis of patients and formulated the corresponding personalized immunotherapy plan. These findings provide a new research direction for the precision treatment of glioma.

## Data availability statement

The datasets presented in this study can be found in online repositories. The names of the repository/repositories and accession number(s) can be found in the article/**Supplementary Material**.

## Ethics statement

Ethical approval was not required for the studies on humans in accordance with the local legislation and institutional requirements because only commercially available established cell lines were used. The animal study was approved by the Animal Care Committee of the First Affiliated Hospital of Shandong First Medical University. The study was conducted in accordance with the local legislation and institutional requirements.

## Author contributions

BW: Writing – original draft. ZW: Writing – original draft. YL: Validation, Writing – review & editing. ZS: Validation, Writing – review & editing. ZL: Formal analysis, Writing – review & editing. HF: Formal analysis, Writing – review & editing. RZ: Conceptualization, Supervision, Writing – review & editing. TX: Conceptualization, Funding acquisition, Writing – review & editing.
