# Supplementary material for: TRIM56: a promising prognostic immune biomarker for glioma revealed by pan-cancer and single-cell analysis
Source: Front Immunol. 2024 Jan 29;15:1327898. doi: 10.3389/fimmu.2024.1327898 (PMC10859405; doi:10.3389/fimmu.2024.1327898)
Supplement: Supplementary file 1 [file DataSheet_1.docx]

Supplementary Material


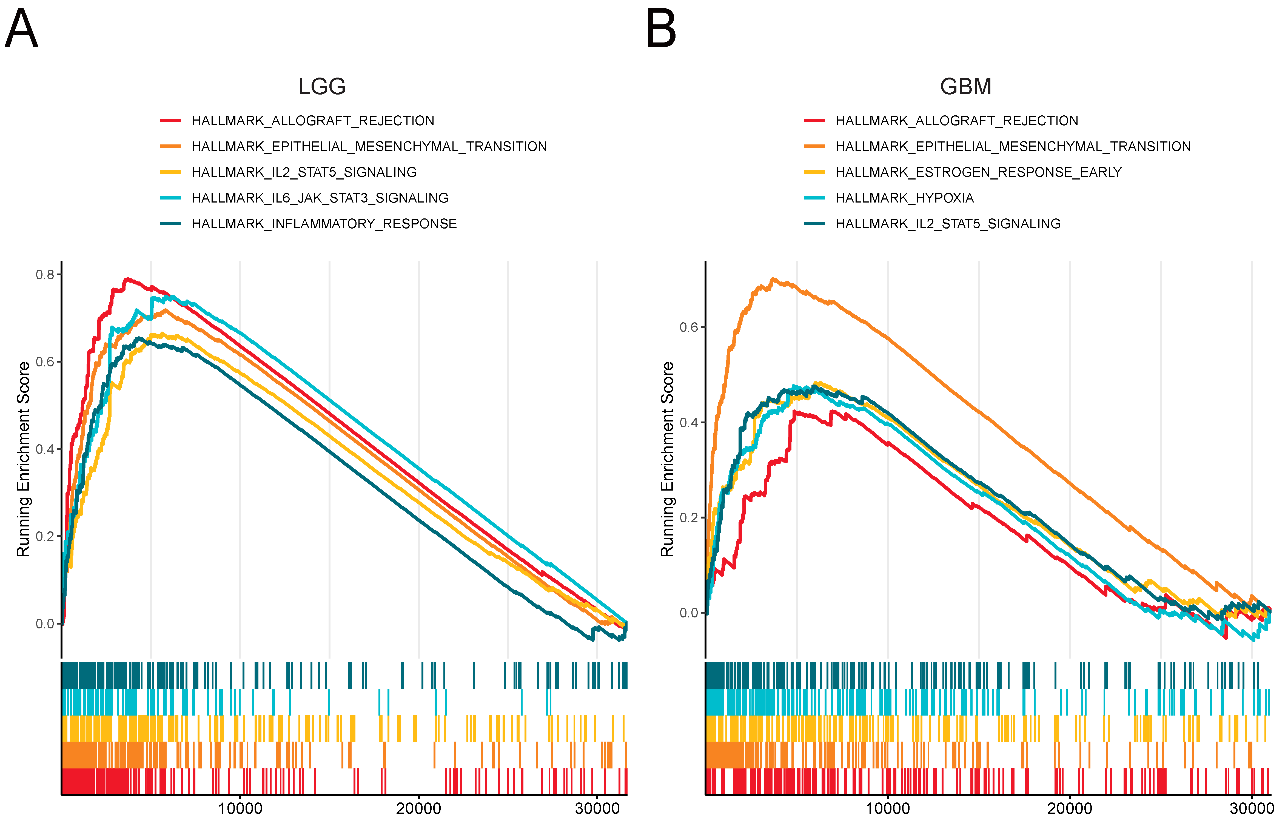


**Supplementary Figure 1.** Gene set enrichment analysis (GSEA) for TRIM56 of the top 5 enriched pathways in LGG (A) and GBM (B).


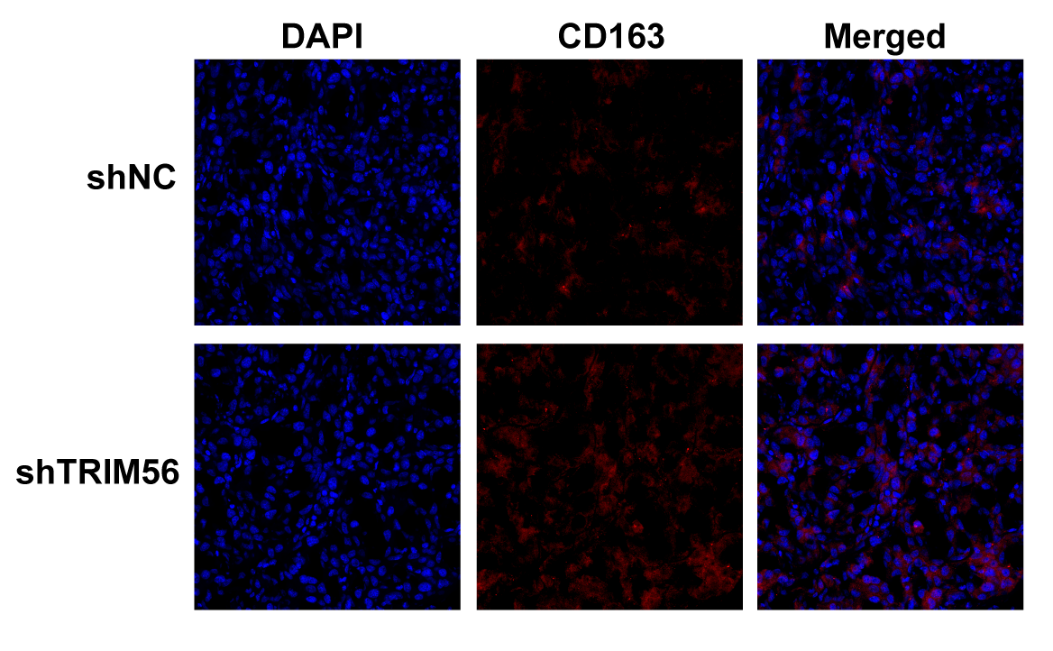


**Supplementary Figure 2.** Overexpression TRIM56 promotes the polarization of M2 macrophages. Immunofluorescence for CD163 expression to detect M2 macrophages in glioma tissue from control and TRIM56 overexpression mice.

**
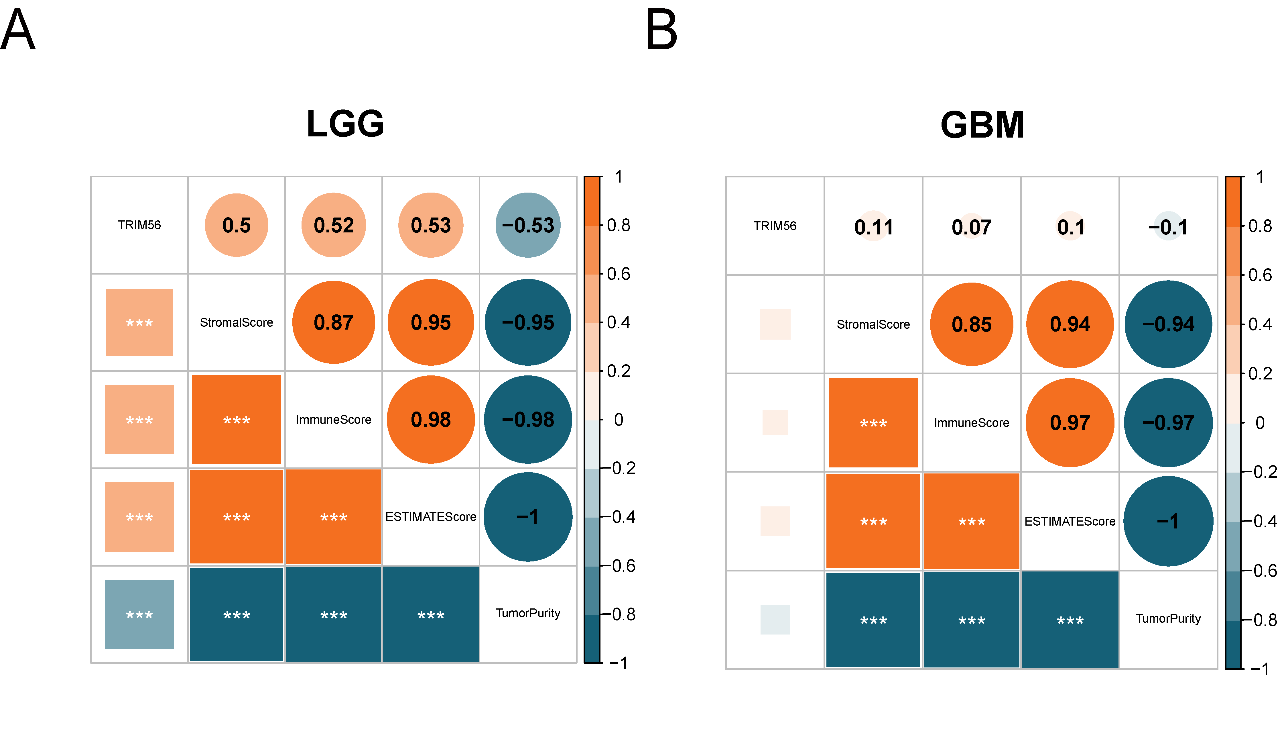
**

**Supplementary Figure 3.** Correlation between TRIM56 expression levels and immune infiltration levels in LGG (A) and GBM (B).**
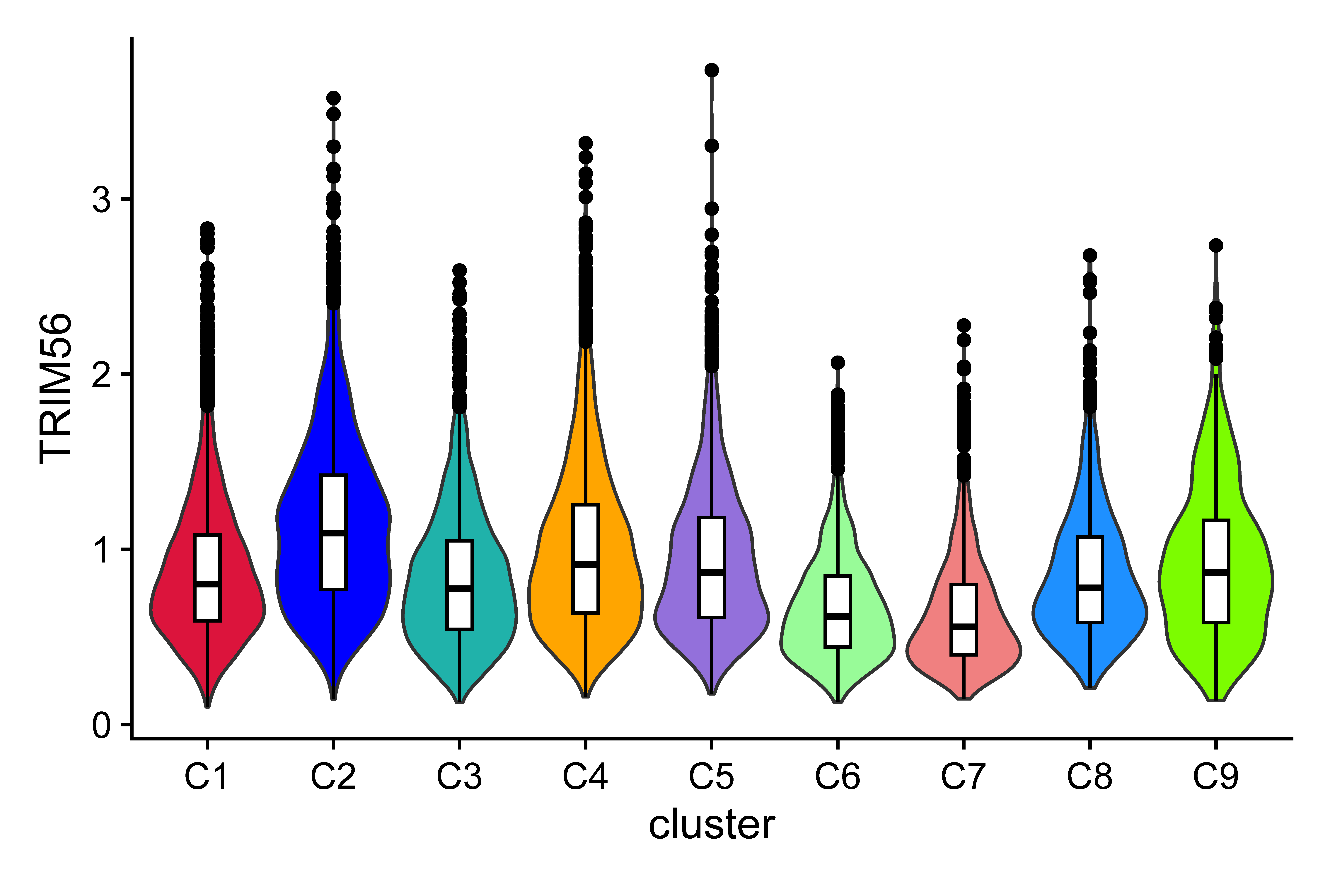
**

**Supplementary Figure 4.** Expression of TRIM56 in different glioma tumor cell subsets.


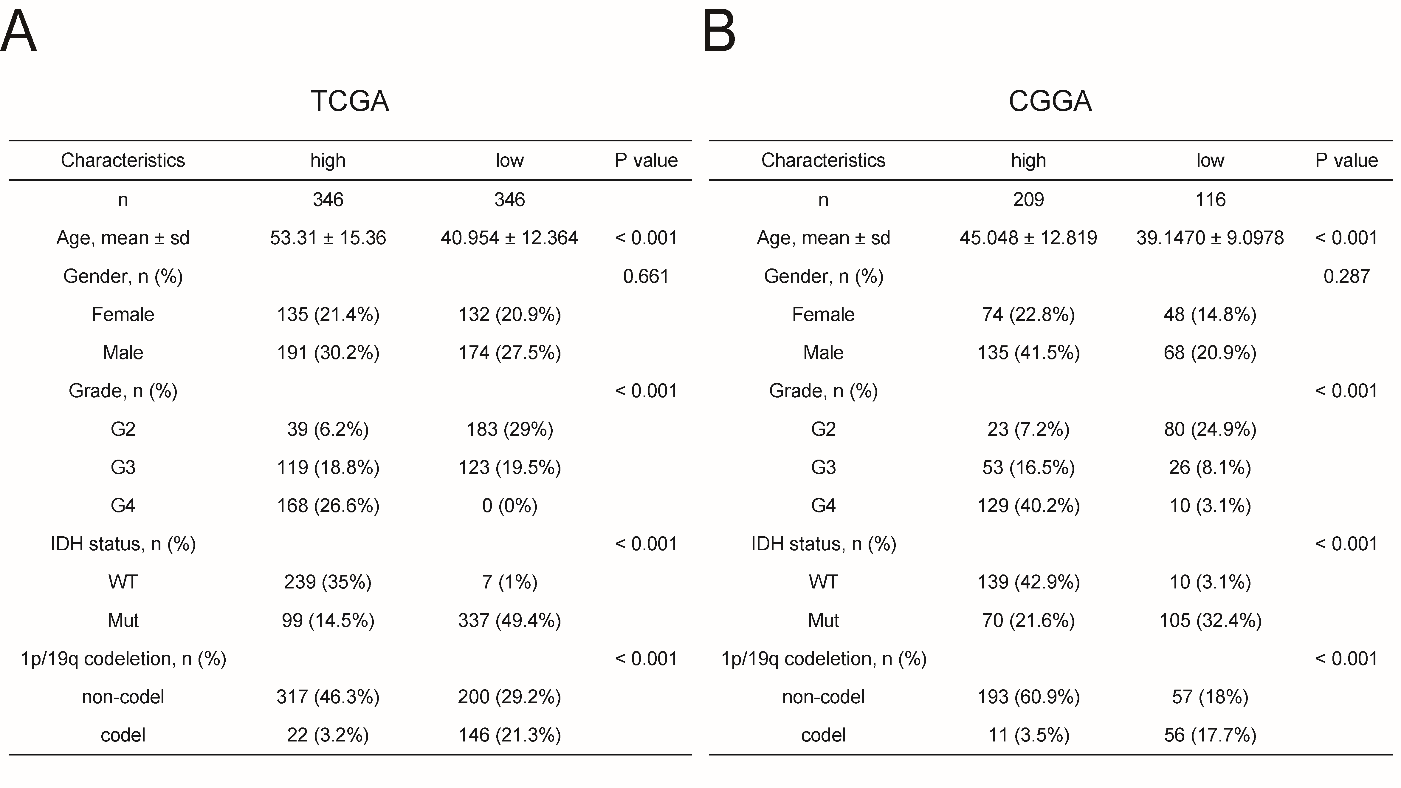


**Supplementary Table 5.** Clinical characteristics of the glioma patients from the TCGA (A) and CGGA (B) cohorts.
